# Melasma Flaring and Unmasking After Treatment With a 1,927-nm Non-ablative Fractional Thulium Fiber Laser

**DOI:** 10.7759/cureus.110920

**Published:** 2026-06-15

**Authors:** Karina Cancel-Artau, Eduardo Michelen-Gómez, Cristina P Gerena-Maldonado, Amara Guerrero-García, Claudia Pérez, Rafael Martín-García, Cristina Brau-Javier

**Affiliations:** 1 Department of Dermatology, University of Puerto Rico, Medical Sciences Campus, San Juan, PRI; 2 Department of Dermatology, Ponce Health Science University, Ponce, PRI; 3 Department of Dermatology, University of Puerto Rico School of Medicine, San Juan, PRI

**Keywords:** flaring, laser therapy, melasma, non-ablative fractional laser, thulium fiber laser, unmasking

## Abstract

Background: The 1,927-nm non-ablative fractional laser (NAFL) has been used as an adjuvant treatment for melasma, though most studies show only temporary improvement. We evaluated the frequency of melasma exacerbations in patients with and without a history of melasma who were treated with a 1,927-nm NAFL for photorejuvenation. Materials and methods: We conducted a descriptive case series based on a retrospective review of patients who received 1,927-nm NAFL (Fraxel Dual) at a dermatology practice in San Juan, Puerto Rico, from June 2018 to June 2023. Charts were examined for prior melasma history, development of new or worsening hyperpigmentation, and the interval between laser exposure and onset of pigmentation changes. Melasma flaring was defined as worsening of preexisting melasma or extension into previously unaffected areas. In contrast, unmasking was defined as new-onset melasma in patients without a prior history of the condition who developed it following laser exposure. Results: Among 116 individuals treated with the 1,927-nm NAFL, 13 patients (11%) developed post-treatment melasma flaring or unmasking. All affected patients were Hispanic women with a mean age of 47 years. Fitzpatrick phototypes included type III in five patients (38%) and type IV in eight patients (62%). The mean time to onset was 13 months for flaring and 17 months for unmasking following NAFL treatment. Conclusions: Lasers that generate dermal heat, including the 1,927-nm NAFL, may precipitate or aggravate melasma in predisposed individuals. Caution is warranted when selecting patients with known risk factors, including Fitzpatrick skin types III-IV and substantial environmental heat and sun exposure. Limitations include retrospective design and a lack of objective severity measures.

## Introduction

Melasma is a chronic, relapsing, acquired hyperpigmentation disorder characterized by symmetric brown-to-gray-brown macules and patches affecting sun-exposed areas of the face, particularly the forehead, malar regions, and chin [1,2]. It disproportionately affects women and individuals with intermediate-to-darker Fitzpatrick phototypes, especially types III-V, who exhibit increased melanogenic responsiveness to ultraviolet (UV) radiation and thermal stimuli [1-3]. Although benign, melasma is therapeutically challenging due to its recurrent nature and persistent melanocyte activation driven by complex epidermal-dermal interactions [1,4].

Epidemiologic estimates of melasma prevalence vary widely across studies and populations, in part due to differences in case definition (self-report vs. clinician-diagnosed), sampling strategies, and geographic distribution. Earlier reports in selected cohorts suggested relatively high prevalence in Latino and Asian populations [1,2,5]. In Brazil, epidemiologic studies describe melasma as common among adult women and frequently associated with intermediate phototypes (III-IV), reinforcing the relevance of ancestry and phototype in risk stratification [2,6]. Given methodological heterogeneity and evolving data sources, prevalence figures must be interpreted in context, and contemporary, population-specific primary epidemiologic data remain limited [2,5].

The pathogenesis of melasma is multifactorial and incompletely understood. Established contributors include cumulative UV exposure, hormonal influences, genetic susceptibility, inflammation, vascular alterations, and environmental heat exposure [3,4]. These factors interact to promote sustained melanocyte stimulation and altered dermal-epidermal signaling, contributing to frequent relapse even after apparent clinical improvement [1,7].

Persistent melanocyte activation, together with dysregulated crosstalk among keratinocytes, fibroblasts, mast cells, and dermal vasculature, underlies the chronicity and treatment resistance characteristic of this disorder [4]. Importantly, melasma is not simply a condition of excess melanin production but rather a complex dysregulation of the entire cutaneous pigmentary unit. Histologic and molecular studies demonstrate upregulation of melanogenic enzymes such as tyrosinase and TRP-1, along with increased expression of pro-pigmentary cytokines, vascular endothelial growth factor, stem cell factor, and inflammatory mediators within lesional skin [3,4].

Additionally, enhanced dermal vascularity, increased mast cell density, basement membrane disruption, and solar elastosis further perpetuate paracrine melanocyte stimulation [4]. These structural and molecular alterations create a self-sustaining microenvironment of melanogenic activation, which explains why monotherapy is often insufficient and why relapse is common following treatment discontinuation [1,7].

Management typically requires a multimodal strategy incorporating topical depigmenting agents, chemical peels, and energy-based devices [7]. Among these, the 1,927-nm non-ablative fractional laser (NAFL) has received FDA clearance for the treatment of actinic keratoses and benign pigmented lesions. It has gained popularity as an adjunctive modality for melasma due to its favorable safety profile, minimal downtime, and documented short-term reductions in MASI scores [8-10].

However, durability of response remains limited. In a long-term follow-up study, recurrence occurred in nearly half of treated patients [11]. Additional studies similarly report transient improvement followed by return of pigmentation over time [12,13]. Beyond early relapse, delayed pigmentary exacerbation following thermal laser exposure has also been described. Q-switched Nd:YAG laser therapy, for example, has been associated with worsening of preexisting melasma and induction of new-onset melasma months after treatment, supporting the concept that heat-based energy delivery may unmask melanogenic susceptibility in predisposed individuals [14]. Despite these observations, limited attention has been given to late-onset flaring or unmasking occurring more than one year after 1,927-nm NAFL treatment, particularly in real-world photorejuvenation settings.

Given the uncertainty surrounding delayed post-laser pigmentary changes, this study aimed to describe the frequency and timing of melasma flaring and unmasking in patients with and without a prior history of melasma who underwent treatment with a 1,927-nm NAFL for photorejuvenation.

## Materials and methods

A descriptive case series was conducted with approval from the Institutional Review Board of the University of Puerto Rico Medical Sciences Campus (protocol #2305107563). Given the retrospective design, the IRB waived the requirement for informed consent.

A retrospective chart review included all patients treated with a 1,927-nm NAFL (Fraxel Dual) at a private dermatology clinic in Puerto Rico between June 2018 and June 2023. Eligibility required periodic follow-up after laser therapy. Patients without follow-up data or with incomplete treatment documentation were excluded.

Demographic characteristics (age, sex, Fitzpatrick phototype), clinical features, and available laser parameters were extracted from medical records. Treatment indications and prior pigmentary history were also documented. Patients who developed melasma flaring or unmasking after laser therapy were identified. Melasma flaring was defined as the worsening of preexisting melasma or its extension into previously unaffected areas. In contrast, unmasking was defined as new-onset melasma in patients without a prior history of the condition who developed it following laser exposure. When available, the date of onset and the temporal relationship to laser treatment were recorded.

Laser parameters (fluence, density, number of passes, and pulse duration) were selected in accordance with standard dermatologic practice and individualized based on Fitzpatrick phototype and treatment indication. During the study period, treatment protocols for 1,927-nm NAFL photorejuvenation at our center followed relatively consistent patterns. However, detailed documentation of specific treatment parameters, including the total number of sessions and exact intervals between treatments, was not uniformly recorded across all charts due to the retrospective nature of the study. Consequently, a granular analysis of parameter-dependent outcomes was not feasible, and this study was not designed to evaluate dose-response relationships.

Prior to treatment, patients underwent a routine dermatologic evaluation, including an assessment of Fitzpatrick's phototype, an indication for treatment, and a review of their relevant medical history. Patients were counseled regarding photoprotection and potential pigmentary risks associated with laser procedures. Treatment was performed using the 1,927-nm NAFL (Fraxel Dual). Topical anesthesia was applied when needed to improve patient comfort during the procedure. Post-procedure recommendations typically included strict photoprotection, avoidance of excessive heat exposure, and the use of gentle skincare products during the recovery period.

Missing data were handled descriptively; cases lacking sufficient documentation of follow-up or pigmentary outcomes were excluded. No imputation methods were applied. Given the descriptive and exploratory nature of this case series, the absence of predefined comparative groups, and the relatively small number of pigmentary events (n = 13), no statistical hypothesis testing was performed. Data were summarized using descriptive statistics to characterize the frequency and timing of events rather than to infer causality.

## Results

A total of 116 patients underwent treatment with a 1,927-nm NAFL. Of these, 13 patients (11%) developed melasma-related pigmentation changes, classified as flaring or unmasking, while the remaining 103 patients (89%) did not develop pigmentary changes during follow-up. All affected patients were female (13, 100%) and Hispanic, with a mean age of 47 years (range, 37-64). Fitzpatrick skin phototypes included type III in five patients (38%) and type IV in eight patients (62%). Except for one patient with an autoimmune disease (1/13, 8%), all affected individuals were otherwise healthy. Full-face treatment was performed in 12 patients (92%). The clinical characteristics of patients who developed melasma flaring or unmasking are summarized in Table 1.

**Table 1:** Clinical characteristics of patients developing melasma flaring or unmasking after 1,927-nm NAFL y: years, F: female, mo: months, NAFL: non-ablative fractional laser

Characteristic	Flaring (n = 8)	Unmasking (n = 5)	Total (n = 13)
Age, mean (range), y	47 (37-64)	46 (38-62)	47 (37-64)
Sex	8 F (100%)	5 F (100%)	13 F (100%)
Fitzpatrick type III	3 (38%)	2 (40%)	5 (38%)
Fitzpatrick type IV	5 (62%)	3 (60%)	8 (62%)
History of melasma	8 (100%)	0 (0%)	-
Full-face treatment	7 (88%)	5 (100%)	12 (92%)
Anatomic distribution	Zygomatic > forehead > upper lip/neck	Zygomatic > forehead > chin/jawline	Zygomatic > forehead > perioral/neck
Mean time to onset, mo (range)	13 (2-27)	17 (8-51)	15 (2-51)

Eight patients (8/13, 62%) with a history of melasma experienced flaring after laser therapy, defined as worsening of existing lesions or development of new lesions. At the time of treatment, six (75%) of these patients had only mild disease. Melasma lesions most frequently involved the zygomatic region, followed by the forehead, upper lip, and neck. The mean time to melasma flaring was 13 months (range, 2-27 months).

Five patients (5/13, 38%) without a prior history of melasma developed unmasking, defined as new-onset melasma after laser treatment. Melasma lesions were most often observed in the zygomatic region, with additional involvement of the forehead, chin, and jawline. The mean time to melasma unmasking was 17 months (range, 8-51 months).

**Figure 1:**
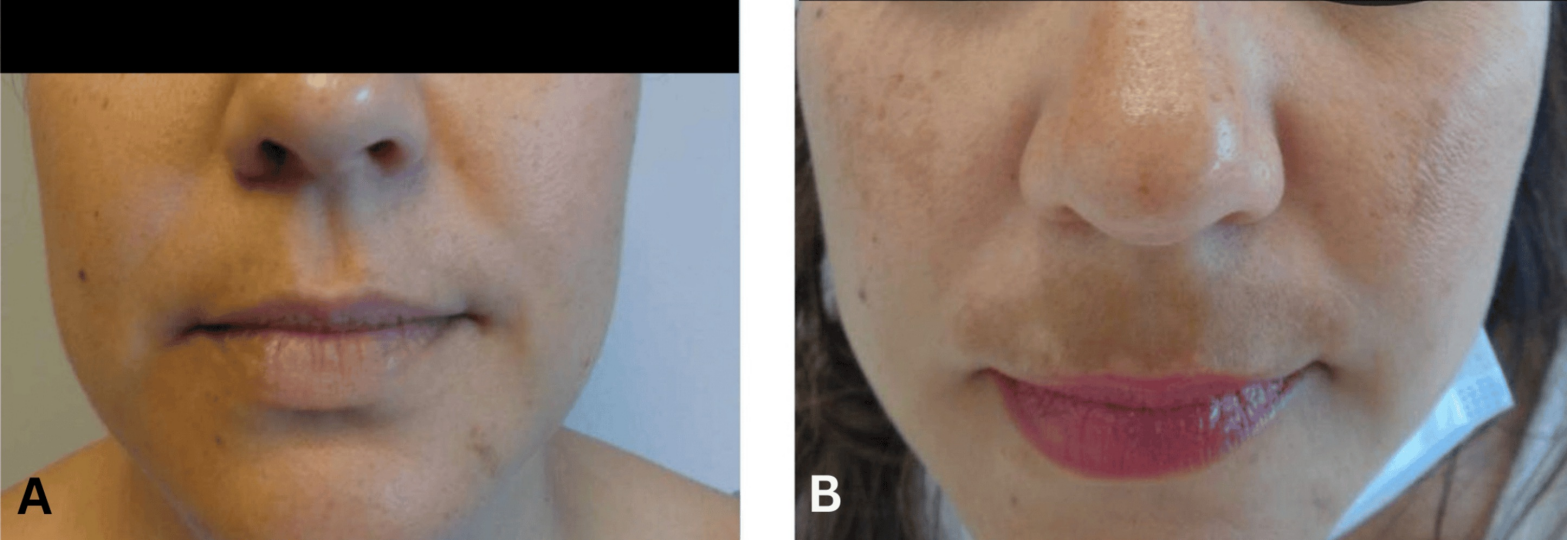
Representative case of melasma flaring after 1,927-nm NAFL treatment (A) Baseline clinical photograph demonstrating preexisting melasma involving the malar/zygomatic cheeks and upper cutaneous lip. (B) Follow-up clinical photograph demonstrating worsening and extension of hyperpigmentation to additional facial areas following 1,927-nm NAFL treatment, consistent with melasma flaring. NAFL: non-ablative fractional laser

A representative patient with post-treatment melasma flaring is shown in Figure 1, demonstrating new-onset facial hyperpigmentation involving the malar/zygomatic cheeks, upper cutaneous lip, perioral region, and chin/jawline after 1,927-nm NAFL treatment.

The remaining 103/116 patients (89%) did not develop pigmentary changes during follow-up. Most demonstrated expected short-term improvement after treatment, and no delayed hyperpigmentation was documented based on available follow-up visits.

## Discussion

The 1,927-nm NAFL generates microscopic columns of controlled dermal injury while preserving the epidermis [8]. The intervening untreated skin facilitates rapid re-epithelialization and dermal remodeling, contributing to its favorable safety profile and shorter downtime compared with fully ablative devices [8]. By design, NAFLs rely on thermal diffusion to induce a wound-healing response, stimulate collagen production, and improve photodamage. However, this same thermal effect may also pose risks for patients predisposed to pigmentary dysregulation.

When laser energy is absorbed by tissue, local heat is generated and subsequently disperses into surrounding structures [15]. In susceptible individuals, this nonspecific thermal spread can precipitate or worsen hyperpigmentation. Epidemiologic observations further support the role of heat in the pathogenesis of melasma. Sarkar et al. demonstrated that patients with sustained exposure to cooking-related heat exhibited more severe disease and higher MASI scores, underscoring the potential role of cumulative thermal stress in pigmentary activation [5].

Several clinical studies have investigated the 1,927-nm NAFL for the treatment of melasma [9-11]. Most reported short-term improvement in MASI scores, particularly within the first 4-12 weeks. In the only study with a mean follow-up of 12 months, Massaki et al. observed significant early improvement, with recurrence during longer-term follow-up [11]. These findings highlight that NAFL may produce meaningful early improvement, but durability is limited, and adverse pigmentary events may occur.

Other studies with shorter follow-up have reported similar trends. Polder and Bruce demonstrated early reduction in pigmentation but noted relapse within months [9]. Ho et al. reported recurrence among treated patients, with worsening MASI scores at follow-up compared with pretreatment values [10]. Lee et al. and Kurmuş et al. also documented transient improvements followed by return of pigmentation [12,13]. Taken together, these findings suggest that NAFL provides only temporary benefit, with a substantial risk of relapse or even rebound pigmentation.

Comparable outcomes have also been reported with other thermal laser devices. Wang et al., in a case-control study of patients treated with Q-switched Nd:YAG laser, observed worsening of preexisting melasma and development of new lesions months after therapy [14]. These delayed pigmentary changes further support the role of heat-based energy in exacerbating or unmasking melanogenic susceptibility.

The findings of the present study are consistent with these observations. In our cohort, 13 patients (11%) developed delayed pigmentary changes, including flaring of preexisting melasma or new-onset disease. Notably, these changes frequently emerged more than one year after laser exposure, suggesting that NAFL-related thermal injury may interact with chronic environmental triggers such as ultraviolet radiation and persistent ambient heat, particularly in tropical climates [5,15].

A substantial proportion of patients were seen only at their one-month post-treatment visit and did not have longer follow-up. This raises the possibility that the true frequency of delayed pigmentation changes may be underestimated. Alternatively, these changes may have developed independently of NAFL exposure, reflecting the chronic and multifactorial nature of melasma [4].

This study has several limitations, including its retrospective design, absence of standardized severity scores such as MASI or mMASI, and a homogeneous cohort composed exclusively of Hispanic females with Fitzpatrick skin phototypes III-IV. Detailed laser parameters, including the total number of sessions, treatment intervals, and complete peri-procedural documentation, were not uniformly available in all charts, limiting the assessment of dose-response relationships and reproducibility. Additionally, standardized pre- and post-procedure clinical photographs were not available for all affected patients. Although a representative case is included to illustrate the observed pigmentary worsening, the retrospective nature of the study limited the availability of uniform photographic documentation across the cohort. While these limitations restrict generalizability, the findings highlight a population at particularly high risk for pigmentary complications.

From a clinical standpoint, these findings emphasize the need for cautious use of NAFL in patients at risk for pigmentary dysregulation. Individuals with preexisting melasma or Fitzpatrick skin phototypes III-IV should be counseled regarding the potential for delayed flares, which may occur months to years after treatment. While NAFL can provide early cosmetic improvement, its benefits must be weighed against the risk of long-term pigmentary instability. In higher-risk populations, more conservative approaches or alternative modalities with less thermal diffusion may be preferable [16].

## Conclusions

This case series suggests that the 1,927-nm NAFL may be associated with delayed melasma flaring or unmasking in susceptible individuals. These findings highlight the potential role of heat-based energy exposure in pigmentary instability, particularly in patients with Fitzpatrick skin phototypes III-IV or living in high-heat environments. Careful patient selection, counseling, and long-term follow-up should be incorporated when using NAFL in populations at risk for melasma.
